# Discovery of New Molecular Subtypes in Oesophageal Adenocarcinoma

**DOI:** 10.1371/journal.pone.0023985

**Published:** 2011-09-23

**Authors:** Daniela Berg, Claudia Wolff, Rupert Langer, Tibor Schuster, Marcus Feith, Julia Slotta-Huspenina, Katharina Malinowsky, Karl-Friedrich Becker

**Affiliations:** 1 Institute of Pathology, Technische Universität München, Munich, Germany; 2 Institute of Medical Statistics and Epidemiology, Technische Universität München, Munich, Germany; 3 Department of Surgery, Technische Universität München, Munich, Germany; Roswell Park Cancer Institute, United States of America

## Abstract

A large number of patients suffering from oesophageal adenocarcinomas do not respond to conventional chemotherapy; therefore, it is necessary to identify new predictive biomarkers and patient signatures to improve patient outcomes and therapy selections. We analysed 87 formalin-fixed and paraffin-embedded (FFPE) oesophageal adenocarcinoma tissue samples with a reverse phase protein array (RPPA) to examine the expression of 17 cancer-related signalling molecules. Protein expression levels were analysed by unsupervised hierarchical clustering and correlated with clinicopathological parameters and overall patient survival. Proteomic analyses revealed a new, very promising molecular subtype of oesophageal adenocarcinoma patients characterised by low levels of the HSP27 family proteins and high expression of those of the HER family with positive lymph nodes, distant metastases and short overall survival. After confirmation in other independent studies, our results could be the foundation for the development of a Her2-targeted treatment option for this new patient subgroup of oesophageal adenocarcinoma.

## Introduction

In the last 10 to 20 years, the incidence of adenocarcinomas arising in the oesophagus has increased faster than that of any other malignancy in the Western world [1]–[4]; in fact, oesophageal adenocarcinoma is currently the seventh most frequent cause of cancer mortalities in these populations [5]. Most patients have a poor prognosis with a 5-year overall survival rate of approximately 25% and unfortunately, this disease is often not diagnosed until it has reached the advanced stages and the tumour has metastasised. Thus, multimodal treatment approaches with preoperative cisplatinum/5-fluorouracil-based chemotherapy or radio-chemotherapy followed by resection are commonly used to improve the survival of patients with oesophageal adenocarcinoma [6]. Recently, some studies have demonstrated a survival benefit for patients that receive peri-operative chemotherapy compared with those that undergo surgery alone for these tumours [7], [8]. However, only 30% to 50% of these patients respond to chemotherapy. For this reason, targeted therapies are in high demand.

Therefore, the focus of this manuscript is on the identification and analysis of new protein signatures for the optimisation of therapy selection to avoid inefficient therapy, toxic side effects and high costs.

In the last few years, the reverse phase protein array (RPPA) has been shown to be valuable for the molecular classification of tumours and the accurate assessment of prognoses and treatment responses. RPPA represents a new high-throughput technology that monitors changes in protein expression over time, before and after treatments, between disease and non-disease states and between responders and non-responders [9].

In this study, we analysed the expression profiles of 17 cancer-related signalling pathway molecules in a series of 87 formalin-fixed and paraffin-embedded (FFPE) oesophageal adenocarcinoma tissue samples by RPPA. We correlated the protein expression patterns with specific clinicopathological parameters and overall patient survival and found that high levels of HER2 and low levels of p-HSP27^(Ser15)^ are correlated with an increased risk of death in patients with oesophageal adenocarcinoma. These findings may assist in the optimisation of patient-specific therapy selection and should provide a basis for the development of new and more effective treatment options for these patients.

## Results

### Unsupervised hierarchical cluster analysis of 87 oesophageal carcinomas

**Figure 1:**
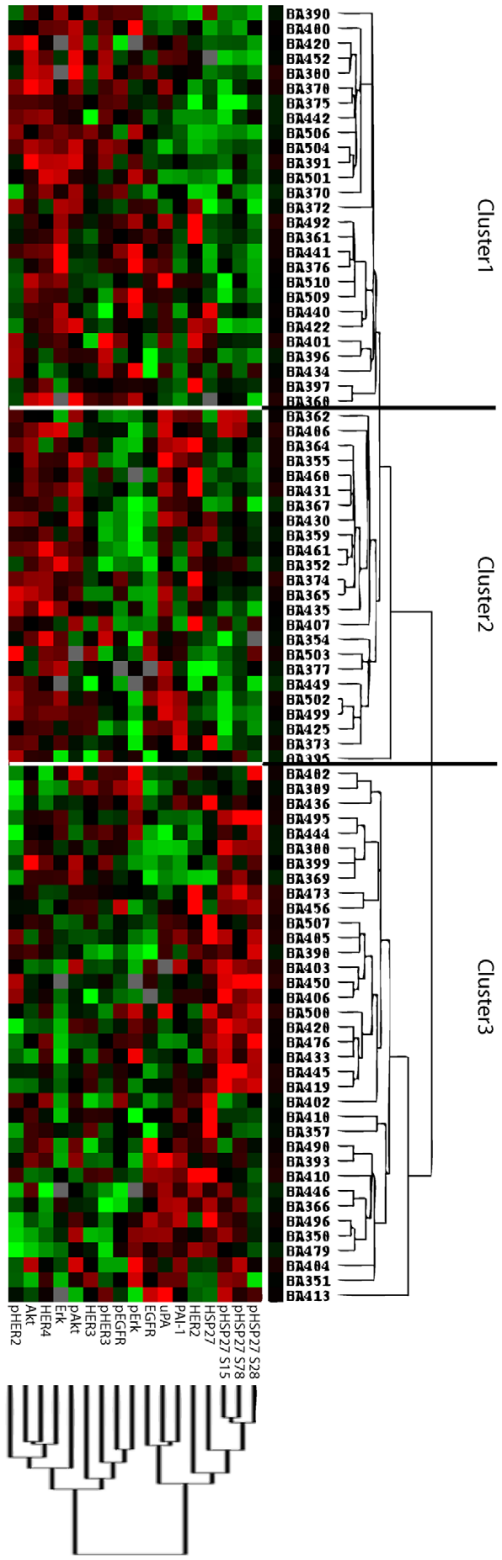
Hierarchical cluster analysis of 87 oesophageal adenocarcinoma cases according to the expression of 17 signalling molecules.

HER2 is a proto-oncogene that is frequently over-expressed in breast cancer and has also recently been found to be over-expressed in carcinomas arising from Barrett's oesophagus and to be correlated with poor survival. Thus, we analysed the proteins of the HER-family (EGFR, p-EGFR, HER2, p-HER2, HER3, p-HER3, HER4) together with other proteins that are known to play important roles in general tumour progression (Akt, p-Akt, Erk, pErk), especially in breast cancer (uPA, PAI-1). In addition, we examined the expression of the heat shock protein HSP27 and its phosphorylated forms (Ser15, Ser78, Ser82) to assess whether the observed link of HSP27 and HER2 in breast cancer cases is also true in oesophageal carcinoma. Using RPPA, we analysed the expression levels of these 17 proteins in 87 oesophageal adenocarcinomas. To obtain deeper insight into the relationships between these proteins and reveal potential overlaps in their signalling pathways, we performed unsupervised hierarchical clustering to generate a depiction of the tumour-specific protein network. As shown in **Figure 1**, the patient cluster formation revealed three groups with different expression profiles. In Cluster 3, a significantly lower amount of tumours were lymph node (24% vs. 64%; p<0.05) or distant metastases positive (0% vs. 13%; p<0.05) compared to combined Cluster 1 and 2. However there were no significant differences in the distribution of relevant clinical parameters (TNM-classification, grading and Lauren's classification) between Cluster 1 and 2.

### Association of heat shock protein 27^(Ser15)^ and HER2 expression and survival

**Figure 2:**
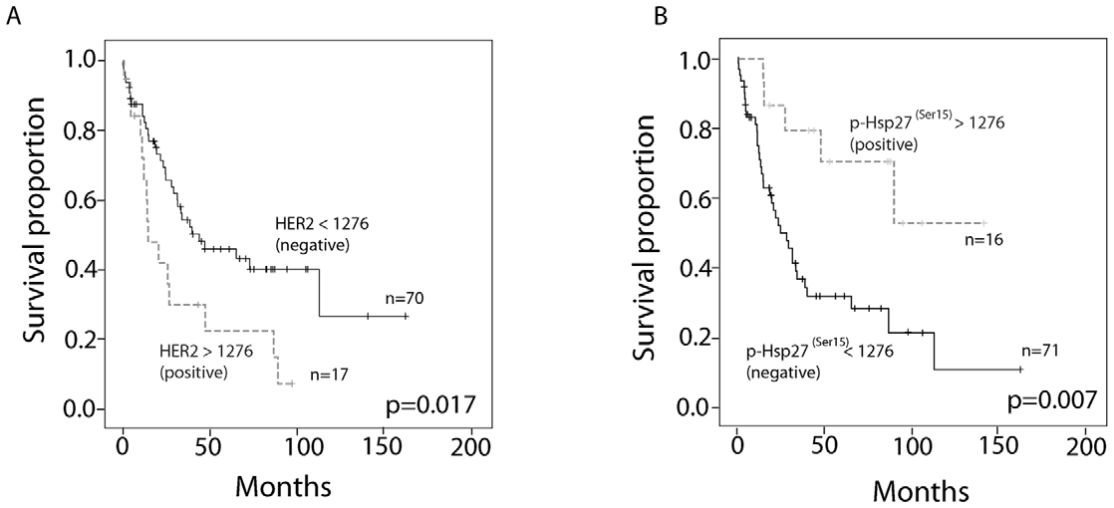
Kaplan-Meier survival curves for patients with A) high versus low HER2 protein expression levels (MicroVigene Signal intensity cut-off level of 1276) and B) high) versus low p-HSP27^(Ser15)^ expression levels (MicroVigene Signal intensity cut-off level of 1316). Statistical significance of differences between groups was evaluated with the log-rank test.

Next, we wanted to determine if some of the analysed proteins had a greater prognostic value in patients with oesophageal adenocarcinoma than others. Thus, we analysed all 17 of the signalling proteins using maximally selected log-rank statistic method [10] of quantitative protein expression in combination with Cox-regression, which allow for optimised risk prediction. Only proteins that showed both, a significant log-rank test for the optimal cut-off value and a significant test for the Cox regression coefficient of the corresponding continuous variable were seen as reliable. Optimal cut-off values could be established for HER2 and p-HSP27^(Ser15)^ (1316 MicroVigene signal intensity points (MVS) for p-HSP27^(Ser15)^ and 1276 MVS for HER2, respectively; see **Figure S1**). HER2-negative patients that were characterised by MVS values of less than 1276 had median survival times of 38.9 months, whereas it was 29.5 months for patients with MVS values of greater than 1276 (p = 0.017; log-rank test, **Figure 2A**). Interestingly, patients with high p-HSP27^(Ser15)^ levels (MVS>1316) also showed prolonged survival (survival rates of 0.7 at 50 months compared with 0.38 for patients with low p-HSP27^(Ser15)^ expression levels; p = 0.007, log-rank test **Figure 2B**). A multivariate Cox regression analysis revealed that HER2 and p-HSP27^(Ser15)^ are prognostic factors for survival independent of those that have already been established, such as depth of tumour invasion (UICC pT category), presence of lymph node metastases (UICC pN category) and presence of distant metastases at the time of resection (UICC cM category) (**see** **Table 1**). This relationship is also illustrated by a nomogram in **Figure S2**.

**Table 1:** Multivariate Cox regression analyses for p-HSP27^(Ser15)^, HER2, pT, pN and cM.

			95%-CIHazard Ratio*		
	coeff	hazard ratio	lower	upper	p-value
p-HSP27^(Ser15)^ **continuous**	−0.0777	0.93	0.88	0.97	**0.0007**
HER2 **continuous**	0.0328	1.03	1.01	1.06	**0.0021**
pT2 vs. pT1	12.761	3.58	1.22	10.54	**0.0204**
pT3 vs. pT1	0.9942	2.70	0.95	7.72	0.0635
pN1 vs. pN0	0.6779	1.97	0.87	4.47	0.1051
cM1 vs. cM0	0.5587	1.75	0.67	4.55	0.2528

			95%-CIHazard Ratio*		
	coeff	hazard ratio	lower	upper	p-value
HSP27Ser15 neg vs. pos†	1.5029	4.49	1.35	15.00	**0.0145**
HER2 pos# vs. neg	0.6757	1.97	1.01	3.82	**0.0465**
pT2 vs. pT1	1.0864	2.96	1.02	8.60	**0.0457**
pT3 vs. pT1	0.8143	2.26	0.77	6.63	0.1387
N1 vs. N0	0.7399	2.10	0.89	4.92	0.0894
M1 vs. M0	0.5089	1.66	0.67	4.13	0.2727

*Risk ratios (hazard ratios) and their lower and upper 95% confidence limits apply for the units of measure in question.

† p-HSP27^(Ser15)^ positive >1316.

# HER2 positive >1276.

Statistically significant p-values are marked in bold letters. pT: tumour status as determined in pathology; pN: lymph node status as determined in pathology; cM: occurrence of distant metastases as determined by attending physician.

### Narrowed, unsupervised hierarchical cluster analysis of 87 oesophageal adenocarcinomas

Because p-HSP27^(Ser15)^ and HER2 showed prognostic value to the clinical outcome for oesophageal adenocarcinoma patients, we performed a second hierarchical cluster analysis of the investigated members of the HSP27 group (HSP27; p-HSP27^(Ser15)^; p-HSP27^(Ser78)^; p-HSP27^(Ser82)^) and the HER family proteins (EGFR, HER2, HER3, HER4, pEGFR, p-HER2, p-HER3).

**Figure 3:**
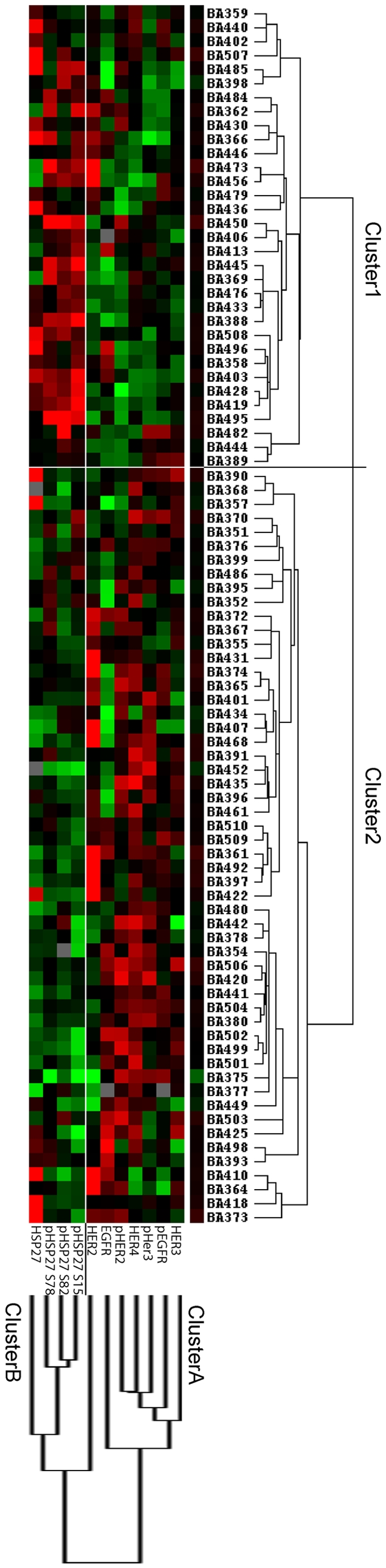
Narrowed hierarchical cluster analysis. The 87 oesophageal adenocarcinoma cases were clustered according to the expression of the investigated members of the (p)HSP27 group and the HER family. Two tumour clusters could be found. Cluster 1 was composed of mainly low HER2-family and high (p)HSP27-group expressing cases, whereas Cluster 2 showed a reverse pattern. Cluster colour key: Red – up-regulated; green – down-regulated; black – unchanged; grey – missing.

**Figure 4:**
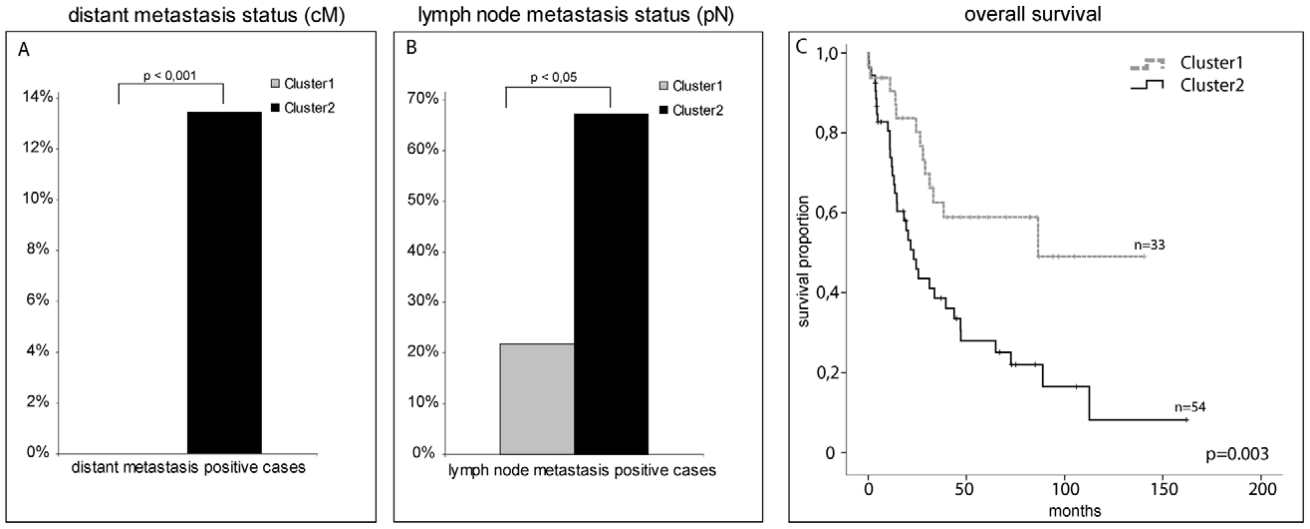
Illustration of the correlation of the obtained clusters with lymph node metastasis status, distant metastasis status and overall survival. Cluster 1, which mainly consisted of low HER2 family and high (p)HSP27 group-expressing cases was comprised of few pN (21%) and no cM (0%) positive cases, whereas Cluster 2 (high HER2 family and low (p)HSP27 group) was comprised of significantly more pN- and cM-positive cases (67% and 15%, respectively). Consistent with these data, patients in Cluster 1 had significantly better overall survival times than Cluster 2.

Two clusters (Cluster A and B) could be distinguished by their protein expression patterns. Cluster A consisted of proteins from the HER family, whereas Cluster B was comprised of HSP27 and its phosphorylated forms (abbreviated as “(p)HSP27”). Two clusters, named Clusters 1 and 2, could be identified from the patient samples (see **Figure 3**). Cluster 1 was composed of tumours that over-expressed (p)HSP27 group proteins and showed low expression levels of HER family proteins, whereas Cluster 2 showed the opposite profile (high levels of proteins from the HER family and low levels of those from the (p)HSP27 group). There were significant differences in relative frequency of distant metastasis (0% vs. 15%) as well as positive lymph nodes (21% vs. 67%) in favour of Cluster 1 (**Figure 4A+B**; For an overview of the composition of the clusters and the p-value of all tested clinical parameters between the two clusters see **Table 2**.). Accordingly, a better survival was observed for cases of Cluster 1 (**Figure 4C**). Therefore, considering p-HSP27^(Ser15)^ and HER2 as prognostic factors, cluster analysis strengthen evidence that low levels of (p)HSP27 are associated with poor patient prognosis, whereas cases showing low levels of HER family proteins have a higher probability of prolonged overall survival.

**Table 2:** Patient and disease characteristics related to the collective used in this study and to the two found clinical relevant clusters.

Factor	No. of patients (%) collective	No. of patients (%) Cluster 1	No. of patients (%) Cluster 2	p-value
**Total**	87 (100)	33 (100)	54 (100)	
**Age**				
mean	63	63	64	
range	33–82	33–82	38–80	0.514
**Gender**				
female	8 (9)	2 (6)	6 (11)	
male	79 (91)	31 (94)	48 (89)	0.513
**pT category**				
pT1	29 (33)	14 (42)	15 (28)	
pT2	19 (22)	7 (21)	12 (22)	
pT3	39 (45)	12 (37)	27 (50)	0.337
**pN category**				
negative	44 (51)	26 (79)	18 (33)	
positive	43 (49)	7 (21)	36 (67)	3.9*10^−5^
**Metastasis**				
cM0	79 (91)	33 (100)	46 (85)	
cM1	8 (9)	0 (0)	8 (15)	**0.020**
**Grading**				
G1	1 (1)	1 (3)	0 (0)	
G2	41 (47)	16 (49)	25 (46)	
G3	45 (52)	16 (48)	29 (54)	0.414

pT category: depth of tumour invasion; pN category: presence of lymph node metastases; cM category: presence of distant metastases at the time of resection (all classification according to UICC). The p-values for cluster comparison using Chi^2^-test (for age Mann-Whitney U test) are given. Significant differences were found for lymph node and distant metastases status (bold).

## Discussion

Molecular profiling of tumour cell-protein networks in routinely processed, clinical cancer tissue samples will be critical for elucidating the process of cancer development and for response prediction to various therapy regimes and targeted therapeutic approaches. The reverse phase protein array (RPPA) represents an emerging technology that is rapidly becoming a powerful tool for the development of drugs and the signal transduction profiling of cellular material. A big benefit of RPPA lies in its ability to generate a patient specific “map” of signalling networks. The RPPA technology is a new, high-throughput technology that monitors changes in protein phosphorylation over time, before and after treatment, between disease and non-disease states and between responders and non-responders [9]. Moreover, it is suitable for the signal transduction profiling of small numbers of cultured cells or cells that are isolated by laser capture microdissection from human biopsies [11], [12]. After protein extraction, each patient sample is arrayed in triplicate on nitrocellulose-coated slides using a miniature dilution curve. Thus, each analyte/antibody combination can be analysed in the linear dynamic range [9], [13].

In the present study, we have applied this technology to find new protein signatures of oesophageal adenocarcinoma that may place patients into different groups based on their protein expression profiles to improve patient-specific therapy selection. We discovered two new molecular subtypes of oesophageal adenocarcinoma patients (**Figure 3**). The first subtype (Cluster1) is characterised by high (p)HSP27 protein levels (cut-off: 1316), low levels of HER family proteins (cut-off: 1276), negative lymph node status, negative distant metastasis status and prolonged overall survival. In contrast, the second subtype (Cluster 2) is defined by low levels of (p)HSP27 and high levels of HER family proteins. Additionally, this subtype showed positive lymph nodes, positive distant metastasis status and short overall survival times. Furthermore, we were able to identify p-HSP27^(Ser15)^ and HER2 as independent prognostic factors in consideration of established survival predictors (such as depth of tumour invasion, presence of lymph node metastases and presence of distant metastases at the time of resection). The found fact that HER2 over-expression or amplification predicts poor survival has been seen in many cancer types such as breast cancer [14],[15],[16],[17] or oesophageal adenocarcinomas [18], [19], whereas to our knowledge no studies showed an influence of p-HSP27^(Ser15)^ in human tumour tissue. However some previous studies showed that HSP27 over-expression is associated with a good prognosis in squamous cell carcinoma of the oesophagus and endometrial carcinoma [20], [21]. Additionally, low pre-therapeutic HSP27 protein expression was associated with poor tumour responses to neo-adjuvant chemotherapy in oesophageal adenocarcinomas [22].

Although the expression profiles of (p)HSP27 proteins and those of the HER family in oesophageal adenocarcinoma have been studied in the recent years, no study has addressed a possible association between these two molecules. However, in breast cancer patients, the cellular functions of HSP27 that is phosphorylated at various sites (Ser78, Ser82 and Ser15) have been analysed, and it was shown that the phosphorylation of HSP27 at Ser78 but not at Ser15 or Ser82 was significantly correlated with positive HER2 and lymph node status [23]. Most interestingly, Kang *et al.* demonstrated that the suppression of HSP27 by specific siRNA increased the susceptibility of SK-BR-3 to trastuzumab [24]. In our study, we were able to demonstrate that although p-HSP27^(S15)^ and HER2 were independent prognostic factors for survival, their expression levels were interrelated in oesophageal adenocarcinoma.

These findings open up new possibilities for the effective treatment of oesophageal adenocarcinoma patients. One major problem has been that, as mentioned above, oesophageal adenocarcinoma patients with low HSP27 levels are resistant to chemotherapy. However, most of these patients show high HER2 expression levels, which suggest that a HER2-specific therapy, such as trastuzumab, may be optimal for these patients. Additionally, a study by Kang *et al.* [24] showed that the suppression of HSP27 by specific siRNA increased the susceptibility of human breast cancer cells to trastuzumab. Therefore, HER2-targeted treatments could lead not only to fewer side effects but also, more importantly, to higher response rates for low HSP27-expressing tumours. In support of our findings, the ToGA study recently showed that trastuzumab in combination with chemotherapy was effective in the treatment of gastric and gastro-oesophageal junction cancers [25]. However, this type of therapy remains to be validated in oesophageal adenocarcinoma patients.

## Materials and Methods

### Ethics Statement

All patients gave informed written consent, and the study was approved by the Ethics Committee of the Technische Universität München, Munich, Germany.

### Tissue samples

Formalin-fixed, paraffin-embedded oesophageal adenocarcinoma tissues from 87 patients diagnosed between 1990 and 2005 were selected from the archive at the Institute of Pathology, Technische Universität München, Germany. Pathological parameters were defined according to the recommendations of the WHO and UICC TNM classification [26]. The patients received neither neo-adjuvant nor adjuvant treatments. Reference haematoxylin/eosin stained sections of the tissues were histologically verified by an experienced pathologist (RL). Only tissue sections containing more than 80% tumour cells were included in the study. For detailed information about the collective, see **Table 2**.

### Antibodies

**Table 3** lists the proteins analysed and antibodies used in the reverse phase protein arrays. All antibodies were validated by western blot using proteins extracted from formalin-fixed tissues (data not shown, for HER2 and p-HSP27^(Ser15)^ antibodies see **Figure S3**).

**Table 3:** Proteins analysed and antibodies used in this study.

Protein	Antibody	Distributor	Dilution
Akt	#9272	Cell signalling, Danvers, USA	1∶1000
p-Akt^(Ser473)^	#9271	Cell signalling, Danvers, USA	1∶1000
EGFR	#2232	Cell signalling, Danvers, USA	1∶2000
p-EGFR^(Tyr1086)^	ZMD.504	Invitrogen, Carlsbad, USA	1∶5000
ERK	#9102	Cell signalling, Danvers, USA	1∶1000
p-ERK^(Tyr202/204)^	#9101	Cell signalling, Danvers, USA	1∶1000
HER2	#A0485	Dako, Glostrup, Denmark	1∶1000
p-HER2^(Tyr1248)^	#44-900	Invitrogen	1∶1000
HER3	#ab40627	Abcam, Cambridge, UK	1∶200
p-HER3^(Tyr1289)^	#4791	Cell signalling, Danvers, USA	1∶1000
Her4	#4795	Cell signalling, Danvers, USA	1∶1000
PAI-1	AHP1100	Serotec, Oxford, UK	1∶5000
uPA	#ab19893	Abcam, Cambridge, UK	1∶500
HSP27	#2402	Cell signalling, Danvers, USA	1∶1000
p-HSP27^(Ser15)^	#ab39399	Abcam, Cambridge, UK	1∶1000
p-HSP27^(Ser78)^	#2405	Cell signalling, Danvers, USA	1∶1000
p-HSP27^(Ser82)^	#2401	Cell signalling, Danvers, USA	1∶1000

### Protein extraction

The protein extraction was performed as previously described [27]. Briefly, tissue sections were deparaffinised and proteins were extracted using EXB Plus. Tumour tissue (containing at least 80% tumour cells) was microdissected and approximately 0.5 cm^2^ tissue from three 10 µm thick sections were processed in 100 µl of extraction buffer. Protein concentrations were determined using the Bradford protein assay according to the manufacturer's instructions (BioRad, Hercules, CA). Randomly selected lysates were probed for β-actin using a western blot to verify the success of the protein extraction and suitability of the material for the reverse phase protein array analysis. All protein lysates that were analysed showed a clear β-actin band on the western blot.

### Reverse phase protein arrays

Reverse phase protein arrays were generated using the Calligrapher MiniArrayer (BioRad, Hercules, CA) according to the manufacturer's instructions. For a general description of the procedure, see references [28], [29]. In the present study, for every lysate and every dilution (undiluted, 1∶2, 1∶4, 1∶8, 1∶16, buffer), three replicates were applied onto a nitrocellulose-coated glass slide (Grace Bio-Labs, Bend, OR) and 3*6 = 18 data points per sample were obtained. Peroxidase blocking was performed according to the manufacturer's instructions (Dako, Glostrup, Denmark). Immunodetection was performed similar to a western blot and as previously described [30]. For the estimation of the total protein amounts, parallel arrays were stained with Sypro Ruby Protein Blot Stain (Molecular Probes, Eugene, OR) according to the manufacturer's instructions.

### Quantitative protein analysis

The tif images for the antibody-detected slides and Sypro Ruby-stained slides were analysed with MicroVigene 3.5.0.0 (VigeneTech, Carlisle, MA). The MicroVigene signal-intensity points (MVS) were calculated by the integral of a logistic 4-point fit model that was matched optimally to the 3*6 data points that were obtained.

### Statistical analysis

#### Unsupervised hierarchical clustering

Unsupervised hierarchical clustering was performed with the Cluster and TreeView softwares. Following log transformation and centre to median calculations, average hierarchical clustering was performed using the Spearman rank correlation [31].

#### Survival analysis

Statistical analyses were conducted using R Software version 2.11.1 (R Foundation for Statistical Computing, Vienna, Austria). To derive optimal cut-off values of quantitative protein expressions that may allow for useful risk predictions, maximally selected log-rank statistics were used. To consider multiple test issues within these analyses, the R-function ‘maxstat.test’ was employed [10].

In addition, survival rates were estimated according to the Kaplan-Meier method. Statistical comparisons between the different patient subgroups were conducted with the log-rank test. Multivariate survival analyses were performed using the Cox proportional hazards regression for HER2 and p-HSP27^Ser15^ together with the clinical variables such as depth of tumour invasion (UICC pT category), presence of lymph node metastases (UICC pN category) and presence of distant metastases at the time of resection (UICC cM category) including risk ratios and 95% CI.

#### Correlation analysis and group comparison

For continuous variables, bivariate relationship was assessed by using Spearman rank correlation coefficient (r_s_). To ensure that the results were clinically relevant, only moderate consistent correlations of |r_s_|>0.40 were considered. For comparisons of frequency distributions between independent subgroups Chi-square test was performed.

## Supporting Information

Figure S1 **Maximally selected log-rank statistics of quantitative protein expression of all 17 analysed signalling proteins to assess the presence of useful risk predictions.** Although in this univariate categorical analysis statistically significant cut-off values could be determined for HER2, p-HSP27^(Ser15)^, Erk and p-HSP27^(Ser82)^, these results were not confirmed in the Cox-regression analysis for Erk and HSP27^(Ser82)^. Therefore credible risk prediction was only possible for the continuous marked proteins (Her2 and p-HSP27^(Ser15)^). Significance level of the log-rank statistics are marked by the horizontal line. The p-values of Cox-regression are given for HER2, p-HSP27^(Ser15)^, Erk and p-HSP27^(Ser82)^. Cut-off values are marked by the vertical dashed line.
(TIF)

Figure S2 **Illustration of the influence of HER2, p-HSP27^(Ser15)^, pT, pN and cM levels on survival.** High expression levels of HER2, low levels of p-HSP27^(Ser15)^ and high TNM levels resulted in high numbers of total risk-points and therefore short survival probabilities for the patients. The survival probability of a specific patient may be determined as follows: first, the risk points for each parameter (HER2, p-HSP27^(Ser15)^, pT, pN, cM) are to be identified separately. Thus, the value of the parameters must be matched to the risk point chart (e.g., if HER2 was calculated to be 4000, the corresponding risk points would be 45). To get the total risk point value, the risk points of all five of the parameters must be added up. These total risk points are then used to map the estimated survival probabilities by matching them with the survival probability chart (e.g., a total risk point value of 120 would result in an estimated 6 month survival probability of 0.8.) [32], [33].
(TIF)

Figure S3 **Validation of HER2 and p-HSP27^(S15)^ antibodies for use in RPPA.** Both antibodies showed high specificity, which is required for the RPPA analysis. To work with the tissue used in the RPPA analysis both western blots were performed with oesophageal adenocarcinoma FFPE tissue.
(TIF)
