# Supplementary figures and images for: Discovery of New Molecular Subtypes in Oesophageal Adenocarcinoma
Source: PLoS One. 2011 Sep 23;6(9):e23985. doi: 10.1371/journal.pone.0023985 (PMC3179464; doi:10.1371/journal.pone.0023985)

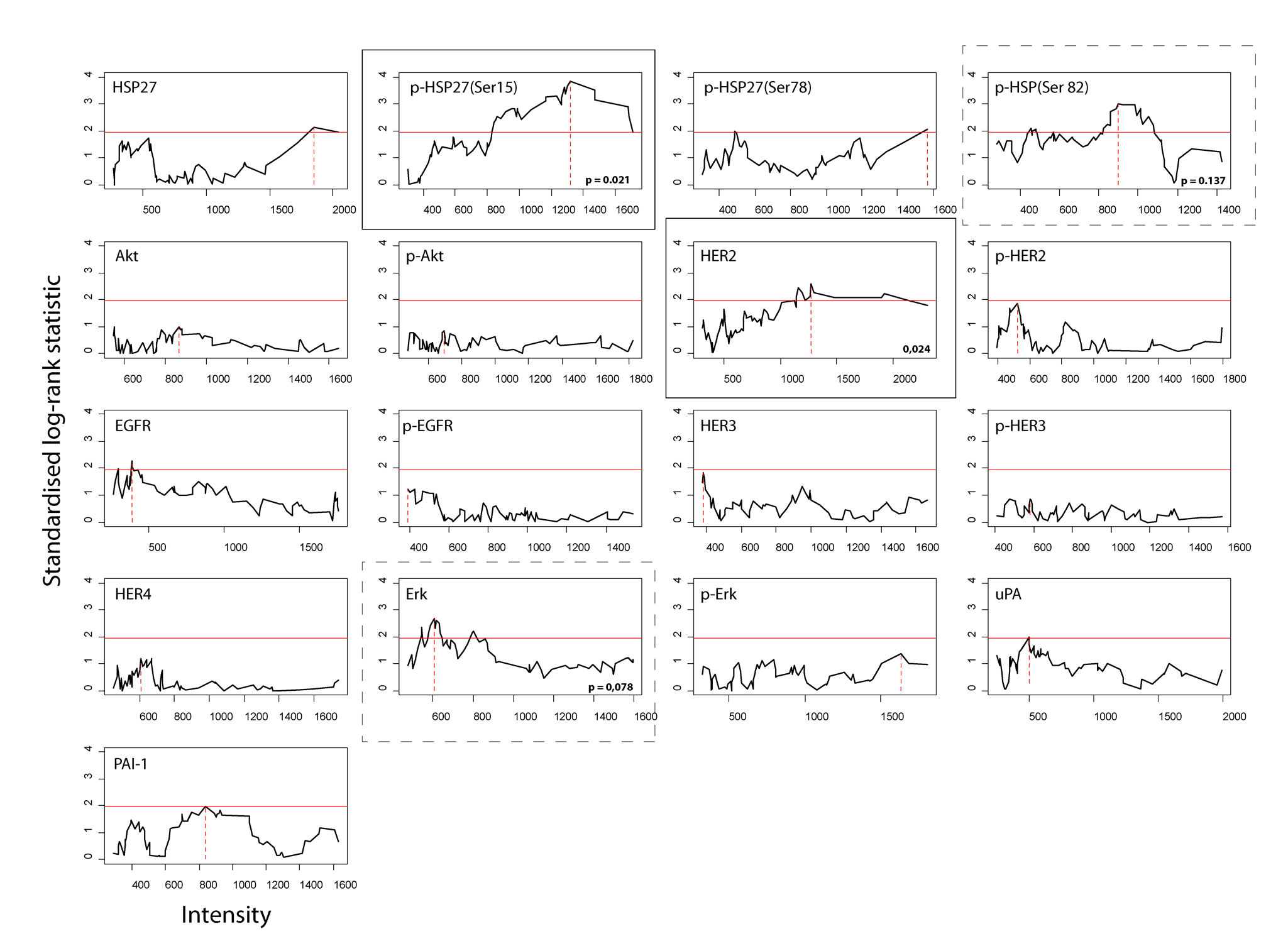

Supplement: Figure S1 — Maximally selected log-rank statistics of quantitative protein expression of all 17 analysed signalling proteins to assess the presence of useful risk predictions. Although in this univariate categorical analysis statistically significant cut-off values could be determined for HER2, p-HSP27(Ser15), Erk and p-HSP27(Ser82), these results were not confirmed in the Cox-regression analysis for Erk and HSP27(Ser82). Therefore credible risk prediction was only possible for the continuous marked proteins (Her2 and p-HSP27(Ser15)). Significance level of the log-rank statistics are marked by the horizontal line. The p-values of Cox-regression are given for HER2, p-HSP27(Ser15), Erk and p-HSP27(Ser82). Cut-off values are marked by the vertical dashed line. (TIF) [file pone.0023985.s001.tif]

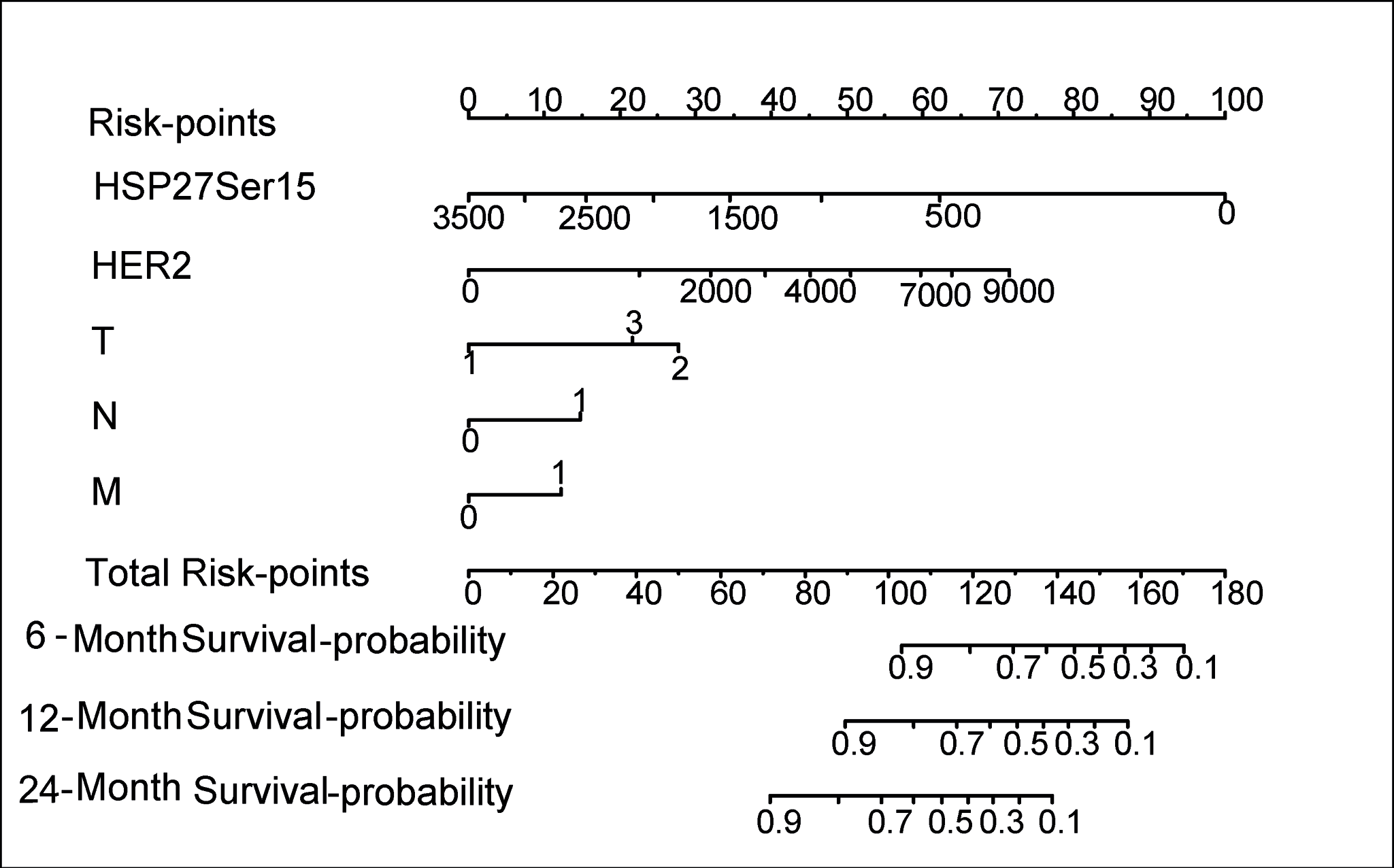

Supplement: Figure S2 — Illustration of the influence of HER2, p-HSP27(Ser15), pT, pN and cM levels on survival. High expression levels of HER2, low levels of p-HSP27(Ser15) and high TNM levels resulted in high numbers of total risk-points and therefore short survival probabilities for the patients. The survival probability of a specific patient may be determined as follows: first, the risk points for each parameter (HER2, p-HSP27(Ser15), pT, pN, cM) are to be identified separately. Thus, the value of the parameters must be matched to the risk point chart (e.g., if HER2 was calculated to be 4000, the corresponding risk points would be 45). To get the total risk point value, the risk points of all five of the parameters must be added up. These total risk points are then used to map the estimated survival probabilities by matching them with the survival probability chart (e.g., a total risk point value of 120 would result in an estimated 6 month survival probability of 0.8.) [32], [33]. (TIF) [file pone.0023985.s002.tif]

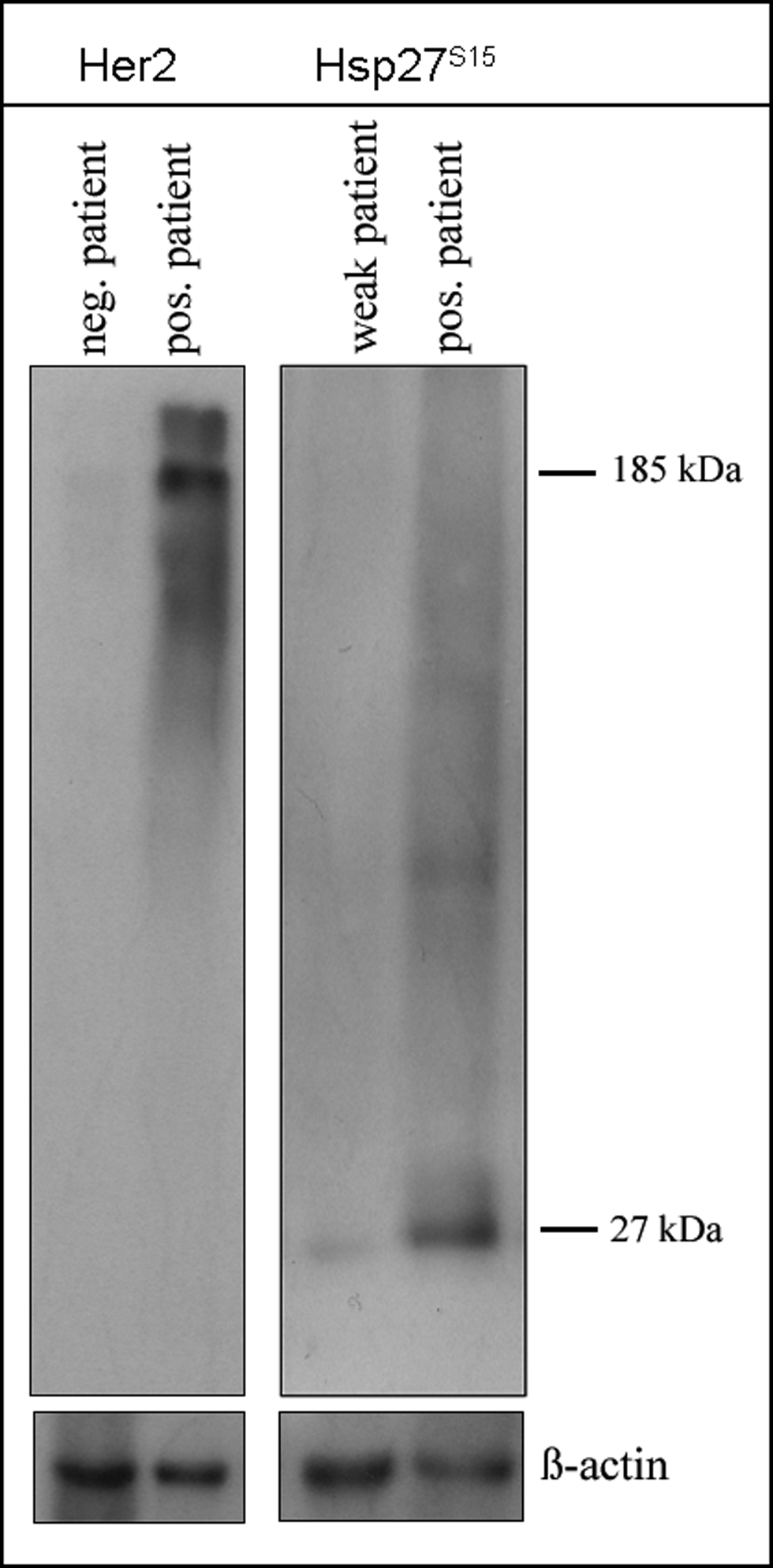

Supplement: Figure S3 — Validation of HER2 and p-HSP27(S15) antibodies for use in RPPA. Both antibodies showed high specificity, which is required for the RPPA analysis. To work with the tissue used in the RPPA analysis both western blots were performed with oesophageal adenocarcinoma FFPE tissue. (TIF) [file pone.0023985.s003.tif]
